# Stereotactic body radiation therapy for early-stage non–small-cell lung cancer in octogenarians and older: an alternative treatment

**DOI:** 10.1093/jrr/rraa027

**Published:** 2020-05-08

**Authors:** Yanping Bei, Naoya Murakami, Yuko Nakayama, Kae Okuma, Tairo Kashihara, Vijay Parshuram Raturi, Hiroyuki Okamoto, Kana Takahashi, Koji Inaba, Hiroshi Igaki, Jun Itami

**Affiliations:** 1 Department of Radiation Oncology, National Cancer Center Hospital, Tokyo, Japan; 2 Department of Radiation Oncology, Ningbo Medical Treatment Center, Lihuili Hospital, China; 3 Department of Radiation Oncology and Particle Therapy, National Cancer Center Hospital East, Kashiwa, Japan

**Keywords:** Octogenarians, non-small-cell lung cancer, stereotactic body radiation therapyr

## Abstract

Surgery is the standard modality for early-stage I–II non-small-cell lung cancer (NSCLC). Generally, patients who are >80 years old tend to have more comorbidities and inferior physical status than younger patients. Stereotactic body radiation therapy (SBRT) may provide an alternative treatment for this group of patients. Here, we report our experience using SBRT to in the management of early-stage NSCLC in patients >80 years old. Patients aged ≥80 years old who were diagnosed with early-stage NSCLC and treated with definitive lung SBRT from January 2000 to January 2018 were retrospectively analysed. Local recurrence-free survival (LRFS), regional recurrence-free survival (RRFS), cancer-specific survival (CSS), progression-free survival (PFS), overall survival (OS) and treatment-related toxicities were analysed for patients >80 years old. A total of 153 patients were included, with a median age of 85 years (range, 80–94). The median follow-up period and OS was 39.8 months (range, 10–101 months) and 76 months, respectively. The 3-year OS, PFS, CSS, RRFS and LRFS were 65.3, 58.0, 75.7, 73.9 and 85.3%, respectively. Radiation pneumonitis grade 0–1, grade 2, grade 3 and grade 4 was observed in 135 (88.2%), 13 (8.5%), 4 (2.61%) and 1 (0.6%) patient(s), respectively. On multivariate analyses, tumor size, pretreatment C-reactive protein (CRP) value, histology and pretreatment physical state were significantly associated with OS. Definitive lung SBRT appears to have high LRFS and OS without causing high-grade radiation-related toxicities in early-stage NSCLC patients who were >80 years old.

## INTRODUCTION

Lung cancer is one of the most common malignancies in the world and it is known that the incidence of lung cancer increases as a population ages [1–3], therefore, treating very old lung cancer patients will become a big common health problem worldwide with the trend of aging populations. According to the Surveillance Epidemiology and End Results (SEER) database in the USA, the median age of diagnosis of lung cancer was reported to be 71 years in 2016 [4]. In individuals ≥70 years old, lung cancer develops two to three times more frequently than in younger individuals and lung cancer is the leading cause of cancer-related deaths in patients aged ≥80 years [5]. With the widespread implementation of low-dose computed tomography (CT) screening programs, lung cancer is more easily found at the early-stage and the incidence of early-stage lung cancer is expected to increase year by year [6]. While patients who were >80 years old accounted for 14% of all lung cancer in 2007, the proportion of patients >80 years old increased to > 20% in 2016 [7].

Japan is one of the fastest aging societies and the situation is similar to that of the USA. The lung cancer incidence in patients ≥80 years in Japan in 2009 was >700 per 10^5^ population [8]. According to a presumption, the life expectancy of Japan in 2065 is projected to reach 84.95 years for men and 91.35 years for women [9], therefore, the number of lung cancer patients >80 years old will be expected to increase further in the future.

Surgical resection is the standard of care for operable early-stage non-small-cell lung cancer (NSCLC), but the standard treatment for elderly patients, especially for those who are >80 years old is controversial. Elderly patients >80 years old generally have a high comorbidity score which relates to higher perioperative mortality and morbidity [10, 11]. The Charlson comorbidity index (CCI) is the most widely used comorbidity index [12]. Wang *et al*. [13] showed that patients with CCI ≥2 had higher perioperative mortality compared to patients with CCI <2. The Lung Cancer Study Group identified increasing age as an independent risk factor for unfavorable mortality in patients undergoing surgery for NSCLC [14]. Recently, there has been an increasing trend in the adoption of lung stereotactic body radiation therapy (SBRT) for early-stage NSCLC, and it has proved to be an appropriate treatment of choice for medically inoperable early-stage NSCLC with high local control rates and with low toxicity [15–22].

Several articles have reported that definite SBRT with a biologically equivalent dose (BED) of ≥100 Gy for early-stage NSCLC patients who were >80 years old was effective with acceptable toxicity rates [23–28]. However, most of these studies about SBRT for early-stage NSCLC in octogenarians and older included only a small number of patients. So far, the largest sample size ever published is in a study with 109 patients with relatively short follow-up periods (median 24.2 months) in which nearly half of the tumors were not histologically confirmed [29]. In the current study, we collected 153 early-stage NSCLC patients in our institution who were >80 years old with definitive lung SBRT. We analysed these patients’ characteristics and clinical outcomes and radiation-related toxicity to investigate the efficiency and tolerance of SBRT in octogenarians and older.

## MATERIALS AND METHODS

### Patients

A total of 153 patients aged ≥80 years who were diagnosed with early-stage (T1-T2bN0M0) NSCLC and treated with definite lung SBRT from January 2000 to January 2018 in our institution were included in this single-center retrospective study. Clinical staging was performed according to the seventh edition of the American Joint Committee on Cancer guidelines (AJCC) [30]. All patients were staged using CT of the chest and positron emission tomography (PET). The patients with hypermetabolic mediastinal nodes on PET imaging were recommended to undergo mediastinoscopy or bronchoscope-guided ultrasound nodal sampling (EBUS) to confirm the pathology. A pretreatment biopsy was performed in all patients except those who were unable to tolerate the biopsy procedure or who resolutely refused the biopsy procedure. The patients who were not biopsy-proven NSCLC were clinically diagnosed using information such as abnormally elevated tumor markers, hypermetabolic lung nodule on PET imaging, or enlargements of a lesion on consecutive CT images. Reasons for referral to SBRT were categorized into patients who were medically inoperable or patients who refused surgery. The information on patient characteristics collected included gender, age, history of smoking, CCI, history of prior lung cancer, history of other cancer in the past, the severity of Global Initiative for Chronic Obstructive Lung Disease (GOLD) [31], pretreatment pulmonary fibrosis biomarkers [mucin-like glycoprotein (KL-6) and surfactant protein D (SP-D)] and pretreatment C-reactive protein (CRP). KL-6 and SP-D are well-known surrogate markers for pulmonary fibrosis and it was reported that positivity of those markers correlated with increased risk for clinically significant pulmonary fibrosis [32]. Tumor characteristics analysed in this study included histology [adenocarcinoma (ACA), squamous cell carcinoma (SCC), undifferentiated NSCLC and tumors not histologically confirmed], tumor size (cm), tumor location and tumor maximum standard uptake value (SUV_max_). Tumor location was categorized into either ‘peripheral’ or ‘central’: central tumors were defined as tumors located within 2 cm of the proximal bronchial tree, or in cases where the planning target volume (PTV) touched the mediastinal or pericardial pleura; other tumors were classified as peripheral [33].

### Treatment

All patients underwent 4DCT for treatment planning in order to account for intra-fraction tumor movement during respiration, with the patient’s body fixed in a custom-made vacuum cushion. For tumors that moved >1 cm in the craniocaudal direction, a respiratory gating system was applied: a fiducial marker that was visible with ultraviolet light was put on the surface of the patient’s abdomen and a respiratory sigmoid curve was generated. No abdominal compression device was used. The definite lung SBRT was delivered using 3D conformal radiotherapy (3DCRT), intensity-modulated radiation therapy (IMRT), volumetric modulated arc therapy (VMAT), magnetic resonance imaging-guided adaptive radiotherapy [0.35 T tri-cobalt-60 magnetic resonance image-guided radiotherapy (MRIgRT) system (ViewRay, Oakwood Village, OH, USA)] or CyberKnife (CK) (Accuray, Inc., Sunnyvale, CA). The technique of SBRT was determined at the discretion of the attending physician based on the site of the disease, size of the solid component of the tumor, range of internal tumor motion during respiration and weather the patient can properly follow the respiratory instructions of the radiation technicians. The clinical target volume (CTV) was equal to the gross tumor volume (GTV), and the internal target volume (ITV) was the sum of GTVs contoured by all different respiratory phases contoured in the 4DCT. The PTV was generated by adding 3–5 mm margins around the ITV. The principle prescribed doses of the peripheral tumor were 10–13.75 Gy per fraction and treated for 4–6 fractions, while for the central tumor the dose delivered was 6–7.5 Gy per fraction and treated for 8–10 fractions. While the prescription doses were delivered to the isocenter before June 2014, they were delivered to the periphery of the PTV after June 2014. An inhomogeneous correction was applied since April 2008. In 3DCRT, 7–12 non-coplanar static portals were used. All fractionated treatments were given once per day, continuously on business days without gaps between fractions. Treatment characteristics analysed included treatment modality, total dose, number of fractions, BED (α/β = 10 Gy), ITV and PTV.

### Follow-up

The first follow-up visit was set 4–6 weeks after SBRT and then every 3 months for the first 2 years, then annually thereafter. Each visit included a comprehensive history and physical examination, CT imaging of the chest and a toxicity assessment according to the Common Terminology Criteria for Adverse Events version 4.0 [34]. ^18^F-fluorodeoxyglucose-positron emission tomography (^18^FDG-PET) scans were obtained only when there was suspicion of disease recurrence and a need for better distinction between post-SBRT fibrosis and recurrence. Patterns of failure were categorized into local, regional and metastatic failure. Local failure was defined as recurrence at the treated site. Regional failure was defined as recurrence in the same lobe of the lung, ipsilateral hilum and mediastinum. Metastatic failure was defined as recurrence in the contralateral lung or any distant site outside of the thorax. Second primary lung tumors were defined according to the criteria of Martini and Melamed [35]. If a lung nodule found after the completion of SBRT fulfilled any one of the following three criteria, it was regarded as a second primary lung cancer: (i) different histologic results from the first tumor, (ii) same histologic results as the index tumor but diagnosed 2 years after the primary tumor, (iii) same histologic results as the index tumor, diagnosed within 2 years of the primary tumor, but located in different lobes or segments, with no positive intervening lymph nodes and no evidence of metastasis.

### Statistical analysis

Local recurrence-free survival (LRFS), regional recurrence-free survival (RRFS), progression-free survival (PFS), cancer-specific survival (CSS) and overall survival (OS) were calculated using the Kaplan–Meier method and the differences were assessed by the log-rank test. PFS was defined as time from last treatment date to tumor progression and censored at the last follow-up date; CSS was defined as the time from last treatment date to death from lung cancer and censored at the last follow-up date; OS was defined as the time from the last treatment date to death and censored at the last follow-up date. Univariate and multivariate Cox regression analyses were performed to determine whether any patient or tumor characteristics and treatment-related variables were predictors of OS. Multivariate analyses were performed for variables with probability (*P*) values < 0.15 in univariate analysis, and when *P* < 0.05 differences were considered statistically significant. All statistical analysis was performed with IBM SPSS Statistics 22.0 (IBM, Armonk, NY, USA).

Written informed consent was obtained from all patients included in this study. This retrospective study was approved by the institutional review board of the XXX Hospital (the approval number was 2017–091) according to the ethical standards laid down in the Declaration of Helsinki.

## RESULTS

### Patients and treatments

A summary of the patient and tumor characteristics of this study is shown in Table 1. A total of 153 patients who met the eligibility criteria of this study were included. Patients’ ages ranged from 80 to 94 years at the time of treatment, with a median age of 85 years. There were 117 males and 36 females. One hundred and nineteen patients had a history of smoking tobacco. All patients underwent a whole-body ^18^FDG-PET scan before treatment; the median SUV_max_ was 4.48 (range of 1.8–19.59). All the patients had a CCI score ≥4 because all the patients included in this study were >80 years old, and 96 (62.7%) patients had a CCI core ≥6. Fifty-two (34%) patients had a prior history of lung cancer and had experienced surgery, which means these patients were may develop another lung cancer thereafter. Moreover, 45 (29.4%) patients had a prior history of other malignancies. The contraindication of resection in the current study included the following reasons: GOLD stage III–IV in patients, CCI ≥ 6 and a history of the lung resection. One hundred and eight patients (70.6%) were medically inoperable, while 45 (29.4%) patients were considered to be medically operable but refused surgery and received SBRT. Fifty-seven (37.3%), 66 (43.1%), 27 (17.6%) and 3 (2.0%) patients were staged as T1a, T1b, T2a and T2b, respectively. As mentioned before, all the patients were N0M0 assessed by PET-CT. Most of the patients were pathologically confirmed except 21 patients who were unable to tolerate or resolutely refused the biopsy procedure. The treatment details are also summarized in Table 1. Two patients whose lesions were large and near to the stomach in the left lower lobe of the lung were delivered SBRT using MRIgART. Thirty-one patients were treated by the CK, 37 using IMRT, 73 using 3DCRT and 10 using VMAT. Almost all the patients had a BED of ≥100 (α/β = 10 Gy) except 7 patients who were given 60 Gy in 10 fractions (BED_10_ = 80 Gy). Among these 7 patients, 3 patients’ tumors were > 5 cm, and in the other 4 patients, the tumor was located near the hilus pulmonis.

**Table 1:** Patient and tumor characteristics

Characteristic and parameters	Number	Value (%)	
Age (years)	Range	80–94	
	Median	85	
Gender	Male	117	76.5%
	Female	36	23.5%
CCI smoking history	4	13	8.5%
	5	44	28.8%
	6	37	24.2%
	7	39	25.5%
	≥8	20	13.0%
History of prior other cancers		45	29.4%
History of prior lung cancer		52	34.0%
Severity of COPD: GOLD score	No COPD	64	41.8%
	Class I	60	39.2%
	Class II	23	15.0%
	Class III–IV	6	4.0%
Pulmonary fibrosis biomarkers	High SP-D	56	36.6%
	High KL-6	31	20.2%
	Both high	18	11.8%
Reason for referral to SBRT	Medically inoperable	108	70.6%
	Refusal of surgery	45	29.4%
Maximum tumor size (cm)	≤1	4	2.6%
	1–2	53	34.6%
	2–3	66	43.1%
	3–4	23	15.1%
	4–5	4	2.6%
	≥5	3	2.0%
Location	Central	30	19.6%
	Peripheral	123	80.4%
Histology	ACA	79	51.6%
	SCC	46	30.1%
	Undifferentiated NSCLC	7	4.6%
	unproven	21	13.7%
Tumor SUV_max_	Range	1.8–19.59	
	Median	4.48	
SBRT total dose (Gy)	Range	42–50	
	Median	48	
BED (Gy)	Range	100–132	
	Median	105.6	
PTV (cm^3^)	Range	4.5–151	
	Median	25.8	
CRP (mg/l)	Range	0.01–4.54	
	Median	0.14	

COPD = chronic obstructive pulmonary disease.

### Survival and failure

**Fig. 1.**
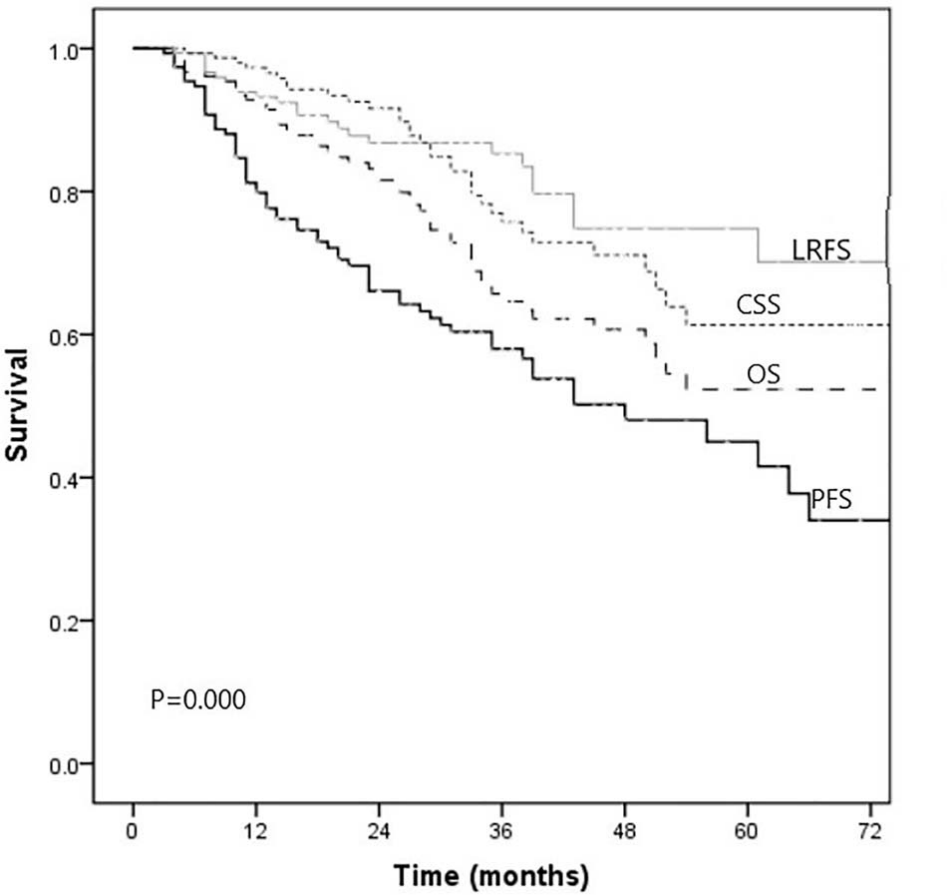
Local recurrence-free survival (LRFS), cancer-specific survival (CSS), overall survival (OS) and progression-free survival (PFS) curves of our SBRT-treated cohort of patients ≥80 years old.

The median follow-up period for survivors was 39.8 months (range, 10–101 months). Among all 153 patients, the median OS was 76 months [95% confidence interval (CI) 48.8–103.1 months], with 3-year OS, PFS, CSS, RRFS and LRFS of 65.3, 58.0, 75.7, 73.9 and 85.3%, respectively. The LRFS, OS, CSS and PFS curves are shown in Fig. 1.

While local failure rate was as low as 15.7%, regional failure was the most frequently seen pattern of failure: 40 patients (26.1%) developed regional failure. Thirteen (8.5%) patients developed distant metastasis. Four out of these 13 patients had both regional failure and distant metastasis. Among 4 patients who developed mediastinal lymph node metastasis, all had tumors larger than 3 cm. During the follow-up time, 8 patients (5.9%) developed second primary lung tumor defined according to the criteria of Martini and Melamed [35], as mentioned previously. Because all the patients included in this study were >80 years old, only 3 patients received systemic chemotherapy after disease progression and all 3 patients had ACA.

### Univariate and multivariate analysis for the factors that correlated with OS

**Fig. 2.**
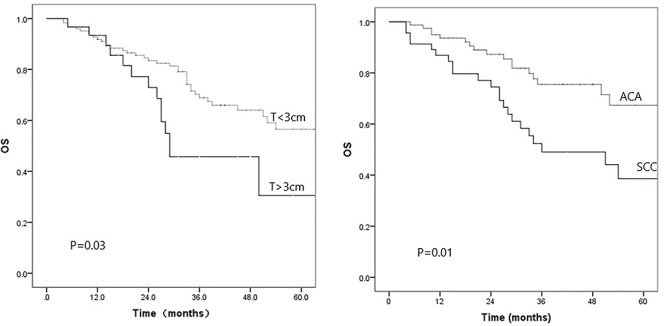
Left: survival curves stratified by tumor size (T) <3 or ≥3 cm. Right: survival curves stratified by the type of histology: adenocarcinoma (ACA) or squamous cell carcinoma (SCC).

Univariate and multivariate analyses of predictive factors of OS are summarized in Table 2. Age, gender, CCI score, tumor size, pretreatment CRP, SUV_max_, history of other tumor, history of primary lung tumor, GOLD stage, the reason for referral to SBRT and pretreatment pulmonary fibrosis biomarker values (SP-D and KL-6) were analysed as clinical variables. In univariate analyses, SUV_max_, tumor size, histology, pretreatment CRP and the reason for referral to SBRT were correlated with OS, with *P* values <0.15. Subsequent multivariate analyses revealed that tumor size, pretreatment CRP, tumor histology and surgically tolerable physical condition were significantly correlated with OS (*P* < 0.05). For patients with tumor size <3 cm, better survival could be expected compared to those with tumor size ≥3 cm (*P* = 0.03) (Fig. 2, left). Another important clinical factor was tumor histology: ACA had a better OS than that of SCC (*P* = 0.01) (Fig. 2, right). Similar univariate and multivariate analyses on LRFS, PFS and CSS were also performed and are summarized in Table 2. It was found that tumor size and histology were the two most important factors associated with the subsequent clinical results.

### Radiation pneumonitis and other treatment-related toxicities

Despite patients’ advanced age and high comorbidity rates, SBRT was well tolerated, and all patients completed the course of SBRT as scheduled. Radiation pneumonitis (RP) was the most concerning complication of lung SBRT. In our cohort, grade 0–1, grade 2, grade 3 and grade 4 RP occurred in 135 (88.2%), 13 (8.5%), 4 (2.61%) and 1 (0.6%) patient(s), respectively. Grade 3 or higher RP toxicity, including dyspnea, cough and pneumonitis, observed in 5 patients, developed 10, 13, 14, 16 and 18 weeks after the first day of SBRT, respectively. One patient who developed grade 4 pulmonary toxicity continued to be oxygen-dependent throughout the 26 month follow-up period. The other 4 patients were oxygen-independent after the conservative radiation pneumonia treatment. The 5 patients who developed Grade 3 or higher grade pulmonary toxicity all had a high level of pulmonary fibrosis biomarkers values (abnormally high SPD and/or high KL6) before SBRT. None of the patients had ≥grade 3 oesophagitis, rib fracture or fatigue related to SBRT. Moreover, there were no other treatment-related toxicities >grade 3 reported at the time of analysis.

### Mortality caused by other than NSCLC

As expected from having a high CCI score in the current cohort of patients, a total of 13 patients died from causes other than NSCLC: 3 patients died of other malignancies, 1 died of mesenteric thrombus, 1 died of acute cardiac disease, 1 died of cerebral hemorrhage, 1 died of rupture of the aortic aneurysm, 4 died of severe aspiration pneumonia and 2 died of unknown causes.

## DISCUSSION

There are only a few randomized clinical trials comparing SBRT to surgery in early-stage NSCLC. The phase III multi-central randomized clinical trial, the ROSEL trial, initiated in 2008 to assess the role of SBRT in patients with operable stage IA lung cancer was terminated early due to slow patient accrual [36]. Another phase III study, called the SABR-Tooth study, which tried to compare SBRT vs surgery in patients with peripheral stage I NSCLC, also failed to meet its recruitment target [37]. Obviously, it is immensely more difficult to perform a randomized clinical trial only for octogenarians and older patients. A retrospective study might be an alternative way to understand SBRT’s efficiency and toxicity in octogenarians and older patients, although the evidence level is lower than for prospective clinical trials. In those past studies that focused on early-stage NSCLC SBRT [16–18, 21, 39], the mean age of patients was generally between 70 and 79 years. On the other hand, the median age of our patient cohort was 85 years, which is much older than those previous papers. To the best of our knowledge, the current study included the largest number of patients who were ≥80 years old, reporting clinical outcomes of lung SBRT in early-stage NSCLC. SBRT has been reported to show excellent local control comparable to that of surgery, with minimal toxicity. The Radiation Therapy Oncology Group (RTOG) 0236 trial showed patients with inoperable NSCLC treated with SBRT had median 4 years OS, 5-year overall survival rate of 40%, high primary tumor control rate of 93% and low distant failure rate of 31% [38]. Some other prospective single-arm trials also showed similar outcomes with high local control rate of ~90% [39, 40]. In the current study, for the 153 early-stage NSCLC patients who were >80 years old, clinical outcomes were not inferior to those previous reports: the median OS was 76 months (95%CI 48.8–103.1 months) and the OS at 1, 2, 3 and 4 years were 92.2, 83.0, 65.3 and 58.7%, respectively. The local control rate was 84.3% (137 out of 153 patients). In addition, the rate of RP related to SBRT in our cohort was <5%, which is not higher than previously reported SBRT results involving patients <80 years old [16–18, 21, 39].

The CCI has been shown to be a prognostic marker for major postoperative complications in patients operated on for primary NSCLC [13, 41]. All patients included in our study had CCI scores ≥4 because all of them were >80 years old, therefore, it is highly expected that they would have high postoperative complication rates and poorer survival if they chose to undergo primary surgery. In our cohort, 93 (62.7%) patients had CCI scores ≥6, which means they were unfit for surgery. Patients in GOLD stage III–IV meant they had insufficient lung function and were contraindicated surgery. Therefore, in our cohort, only 45 (29.4%) patients were considered to be medically operable. SBRT could be another reasonable treatment of choice for this cohort.

Old patients are more likely to develop multiple synchronous or metachronous primary lung cancer than younger patients [42]. It was found in the current study that patients who were >80 years old had a high rate of prior history of lung tumor (34%) and also had a tendency to develop another primary lung cancer after SBRT (5.9%), which is in line with previous findings [42]. In our cohort, 52 patients (34%) had a prior history of lung tumor for lobectomy, and 8 patients had a second primary lung tumor during the follow-up period. In this context, repeated SBRT can be offered to a different part of the lung in selected patients with preserved lung function and could be a reasonable option for such occasions.

In the current study, the multivariate analyses showed that the patients who were medically operable had better survival. Palma *et al*. [21] conducted a matched-pair comparison of SBRT vs surgery and concluded that similar OS outcomes were achieved. The study did not take patients’ or physicians’ preferences for treatment selection into account. Usually, the patients who are treated by SBRT tend to have a higher CCI score and worse performance status than patients who receive surgery. Therefore, it could be stated that if the patients are medically operable a favorable OS could be expected by SBRT similar to surgery. Yu *et al*. [43] used the SEER-Medicare linked database to compare the outcome of SBRT and surgery in old patients in early-stage NSCLC. It was found that the patients who underwent SBRT had a significantly lower rate of early mortality and complications than those who underwent surgery for stage I NSCLC. However, 2 years or more after treatment, patients who underwent surgery tended to have superior oncologic outcomes. Therefore, it could be said that for patients with short life expectancy, SBRT might be the preferable treatment of choice. This conclusion may be suitable for those patients who are octogenarians and older who have a relatively short life expectancy.

**Table 2:** Clinical factors related to overall survival (OS), local recurrence-free survival (LRFS), cancer-specific survival (CSS), and progression-free survival (PFS).

Clinical factors	Univariate analyses		Multivariate analysis	
	Hazard ratio (HR) (95% CI)	*P* value	Hazard ratio (HR) (95% CI)	*P* value
OS				
SUV_max_	1.09 (1.02–1.17)	0.02		
Tumor size (<3 cm vs >3 cm)	2.00 (1.08–3.71)	0.03	1.95 (1.09–3.48)	0.04
CRP	1.58 (1.09–2.29)	0.02	1.87 (1.28–2.78)	0.03
Histology (ACA vs SCC)	2.27 (1.20–4.16)	0.01	2.04 (1.01–4.00)	0.02
Reason for referral to SBRT (refused surgery vs medically inoperable)	1.60 (0.99–3.03)	0.01	1.41 (1.25–4.23)	0.04
GOLD stage (normal–I vs II–III)	1.61 (0.78–3.33)	0.19		
CCI score	1.22 (0.96–1.55)	0.10		
LRFS				
SUV_max_	2.01 (1.16–3.51)	0.01		
Tumor size (<3 cm vs >3 cm)	1.39 (1.01–2.71)	0.03	1.70 (1.15–2.51)	0.01
CRP	1.51 (0.87–2.62)	0.14		
Histology (ACA vs SCC)	1.61 (0.65–3.99)	0.30		
Reason for referral to SBRT (refused surgery vs medically inoperable)	1.46 (0.54–3.91)	0.46		
GOLD stage (normal–I vs II–III)	1.15 (0.64–2.05)	0.64		
CCI score	1.18 (0.84–1.66)	0.33		
CSS				
SUV_max_	1.12 (0.98–1.88)	0.02		
Tumor size (<3 cm vs >3 cm)	1.25 (1.02–2.01)	0.03	1.02 (1.10–1.19)	0.04
CRP	1.12 (0.78–1.60)	0.56		
Histology (ACA vs SCC)	2.72 (1.14–4.83)	0.01	2.46 (1.12–5.40)	0.03
Reason for referral to SBRT (refused surgery vs medically inoperable)	1.09 (0.53–2.23)	0.82		
GOLD stage (normal–I vs II–III)	1.71 (0.77–3.81)	0.19		
CCI score	1.19 (0.92–1.54)	0.18		
PFS				
SUV_max_	1.58 (1.07–2.35)	0.02		
Tumor size (<3 cm vs >3 cm)	1.22 (0.94–1.59)	0.08		
CRP	1.21 (0.91–1.60)	0.19		
Histology (SCC vs ACA)	1.93 (1.10–3.39)	0.02	1.06 (1.08–3.75)	0.04
Reason for referral to SBRT (refused surgery vs medically inoperable)	1.59 (0.80–3.16)	0.19		
GOLD stage (normal–I vs II–III)	1.63 (0.77–3.45)	0.20		
CCI score	1.19 (0.95–1.50)	0.13		

Histology was an independent prognostic factor on OS and CSS in the current study. Patients with ACA histology had a better OS and CSS than that of patients with SCC. The differences in subsequent treatment after disease failure may be the reason for the differences in survival between the two groups because ACA patients had much more subsequent effective systemic treatment such as various targeted agents.

There are several limitations to this study. It was a retrospective study from a single institution with limited follow-up period. One major limitation of a retrospective study is the inherent biases, including the reliance on medical records for accurate information on follow-up. Dose prescription, treatment machine and dose calculation methods were not consistent throughout the study period and different fractionation was applied for tumors larger than 5 cm or centrally located. Although we attempted to document many known variables related to outcomes, including CCI, smoking status and previous malignancy, there are undoubtedly confounding factors that are not accounted for given the retrospective nature of our study.

## CONCLUSION

Definitive lung SBRT resulted in high LRFS and OS with minimal high-grade toxicities in early-stage NSCLC patients >80 years old. SBRT can be an alternative treatment of choice for octogenarians and older patients, regardless of whether they are medically operable or not.

## CONFLICT OF INTEREST

Dr. Itami reports personal fees from HekaBio, other from Kay J, outside the submitted work.

Dr. Igaki reports grants from HekaBio, personal fees from Itochu, outside the submitted work.

Dr. Inaba reports grants from Elekta, outside the submitted work.

Dr. Nakayama reports personal fees from AstraZeneca, outside the submitted work.

The other authors declare that they have no conflict of interest to declare.

## Funding

This study was partially supported by the Japan Agency for Medical Research and Development, (AMED, 17ck0106305h0001), the National Cancer Center Research and Development Fund (26-A-18 and 26-A-28). 
